# The prevalence and predictors of post–intensive care syndrome following cardiac surgery

**DOI:** 10.1016/j.xjon.2025.03.017

**Published:** 2025-03-31

**Authors:** Amanda Rea, Sarah Holler, Rakesh Arora, Rebecca Hottle, Clifford Fonner, Olivia Marx, Rawn Salenger

**Affiliations:** a Division of Cardiac Surgery, Department of Surgery, University of Maryland St Joseph Medical Center, Towson, Md; b Perioperative and Cardiac Critical Care, Harrington Heart and Vascular Institute, University Hospitals, Cleveland, Ohio; c Department of Surgery, Case Western Reserve University, Cleveland, Ohio; d Maryland Cardiac Surgery Quality Initiative, Baltimore, Md; e University of Maryland School of Nursing, Baltimore, Md; f Division of Cardiac Surgery, Department of Surgery, University of Maryland School of Medicine, Baltimore, Md

**Keywords:** cardiac surgery, PICS, post–intensive care syndrome

## Abstract

**Objective:**

Post–intensive care syndrome has been well documented in the general critical care population, but the prevalence of post–intensive care syndrome in the cardiac surgery population remains uncertain. We sought to define the prevalence of post–intensive care syndrome and associated risk factors after adult cardiac surgery.

**Methods:**

Data were collected on 397 consecutive adult patients undergoing cardiac surgery. The patients were surveyed 4 weeks after surgery using the Healthy Aging Brain Care Monitor Self-Report version between June 2022 and June 2023.

**Results:**

Seventy percent of patients reported symptoms consistent with mild (50%) or severe (20%) post–intensive care syndrome. Patients with severe post–intensive care syndrome score were more likely to be female (*P =* .04), to be White (*P =* .03), and to have new dialysis (*P =* .01). Hypoglycemia (*P <* .001) and high Richmond Agitation Sedation Scale score (*P =* .001) were also associated with severe post–intensive care syndrome. Further, a history of diabetes (*P =* .05), depression (*P =* .01), and anxiety (*P =* .01) were more commonly observed in patients with post–intensive care syndrome.

**Conclusions:**

Our study demonstrated a significant prevalence of post–intensive care syndrome after cardiac surgery. We identified female gender, White race, hemodialysis, hypoglycemia, and higher Richmond Agitation Sedation Scale scores as factors associated with increased risk of post–intensive care syndrome.

**Figure:**
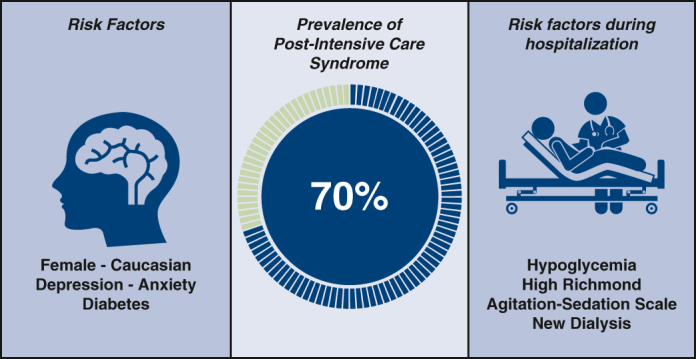
Prevalence and risk factors of patients with PICS after CS.

**Central Message**

PICS occurs in patients after CS and requires more research and awareness within the CS community.

**Perspective**

PICS is well documented in the critical care literature, typically affecting patients after stays in the ICU. It includes physical, cognitive, and psychological symptoms that can persist after discharge. Although patients undergoing CS often experience critical illness and ICU stays, there is limited research focusing on how PICS manifests in this group.

Each year, in the United States there are more than 5 million admissions to intensive care units (ICUs).^1^ Although modern intensive care practice has resulted in significant advances in caring for critically ill patients, ironically with these advances, patients are exposed to numerous traumatic experiences during the course of their care.^2^ Post–intensive care syndrome (PICS) affects approximately 30% to 80% of ICU survivors, with effects lasting up to 5 to 15 years after the hospitalization.^1^^,^^3^ In critical care literature, symptoms such as anxiety, depression, and reduced quality of life can be categorized in the domains of behavioral, cognitive, psychosocial, and physical symptoms.^4^ Collectively, these are described as PICS and have been shown to persist for months to years in ICU survivors. Patients with prolonged length of stay (LOS) and depression after cardiac surgery (CS) have been linked to decreased Health-Related Quality of Life.^5^, ^6^, ^7^ The Repeatable Battery for the Assessment of Neuropsychological Status study showed survivors of critical illness were similar to patients with traumatic brain injury and those who have Alzheimer's dementia.^1^^,^^8^

PICS has been well documented in the general critical care population, but the prevalence of PICS in the CS population remains uncertain. This patient population is increased because they use medications throughout the perioperative process that have been associated with an increased risk of PICS, including benzodiazepines, sedatives, paralytics, and analgesics.^9^ We sought to define the prevalence of PICS and associated risk factors after adult CS. Because of the lack of robust evidence linking PICS and CS, we prospectively analyzed our adult patients undergoing CS.

## Patients

Data were collected on 397 consecutive adult patients who underwent coronary artery bypass grafting (CABG), valve repair or replacement, combined CABG and valve surgery, and open aortic procedures between May 2022 and June 2023. Our program is a tertiary care community program performing approximately 600 CS cases annually. This study was approved by our Institutional Review Board (HP-00091144, 4/17/2020), and requirement for consent was waived. The patients were surveyed 4 weeks after surgery using The Healthy Aging Brain Care Monitor Self-Report version (HABC-M SR), between June 2022 and June 2023. All patients who returned to our office for their 4-week follow-up appointment were included in the study. We included patients at all levels of literacy and languages or with visual or hearing deficits. These patients were provided with assistance devices, if needed.

## Material and Methods

Despite the growing recognition of PICS, there is a lack of validated tools to assist with identification, assessment, and severity. The HABC-M SR is the only validated tool that evaluates all categories of PICS. There is not a CS-specific tool available at this time.^3^^,^^10^ The cognitive section of HABC-M SR involves 6 questions related to memory, orientation, and judgment. The functional section has 11 questions related to activities of daily living. The psychological section has 10 questions related to depression, psychotic, and anxiety symptoms. The patient is asked how many days they experienced symptoms over the past 2 weeks. Scoring is defined as 0 = not at all (0-1 day), 1 = several days (2-6 days), 2 = more than half the days (7-11 days), and 3 = almost daily (12-14 days). The HABC-M SR has not established criteria for correlating weak and strong positive scoring or severity. For the purposes of this evaluation, we defined a score of 0 as no evidence of PICS, a score of 1 to 9 as a weak positive, and a score greater than 10 as a strong positive. The questionnaire was completed in written form at the first postoperative outpatient follow-up appointment. Patients were identified and voluntarily completed the questionnaire. We chose to use the postoperative appointment, so we did not have an increased rate of functional deficits due to being debilitated from surgery. Patients not captured were those in rehabilitation, who were lost to follow-up, or who refused to complete.

Process measures, lengths of stay, discharge status, and risk factors were compared between patients with no PICS, weak positives, and strong positives (Table 1). Metrics were summarized with means and SDs for continuous measures and with proportions for categorical variables. Statistical tests compared strong positives (high scoring surveys) against all others, as well as strong (high scoring) and weak (lower scoring) positives combined versus patients with no PICS. Variables were compared with the *t* test for continuous measures and the chi-square test for categorical variables. Clinical data were retrieved from the electronic medical record.

**Table 1:** Outcome data

PICS survey results: N = 397 patients	No PICS (0 survey points)	Weak positive (1-9)	Strong positive (10+)	*P* value (strong vs others)	*P* value (strong + weak combined vs no PICS)
N	117 (29%)	200 (50%)	80 (20%)		
Hospital LOS (d)	7.65	7.27	8.65	.04	.99
ICU LOS (d)	2.04	2.16	2.60	.13	.40
Mean ventilation time (h)	17.11	15.57	16.12	1.00	.65
Early extubation (<6 h)	0.9%	1.5%	2.5%	.42	.49
Prolonged ventilation (≥24 h)	10.3%	7.0%	15.0%	.06	.76
Discharged home	92.3%	90.0%	83.8%	.06	.23
RASS high	0.58	0.66	1.01	**.001**	.07
RASS low	−0.96	−0.96	−1.15	.15	.66
Depression	3.4%	10.5%	12.5%	.19	**.01**
Anxiety	7.7%	17.5%	16.3%	.59	**.01**
Smoker	28.2%	31.5%	21.3%	.11	.94
Alcohol use	17.1%	14.5%	13.8%	.70	.48
Glucose high	206.09	216.32	223.19	.21	.10
Glucose low	92.56	95.04	86.23	**<.001**	1.00
Sedation/pain/NMBD	12.8%	14.5%	16.3%	.59	.57

Bold indicates stastically significant. *PICS*, Post–intensive care syndrome; *LOS*, length of stay; *ICU*, intensive care unit; *RASS*, Richmond Agitation Sedation Scale; *NMBD*, neuromuscular blockade.

**Table 2:** Outcome data per case type

PICS survey results per case type	No PICS (0 survey points)	Weak positive (1-9)	Strong positive (10+)	*P* value (strong vs others)	*P* value (strong + weak combined vs no PICS)
CAB only: N = 297 patients					
N	81 (27%)	161 (54%)	55 (19%)		
Hospital LOS (d)	7.51	7.23	8.44	.09	.96
ICU LOS (d)	1.82	2.04	2.70	**.03**	.18
Mean ventilation time (h)	14.04	14.06	16.56	.49	.84
Early extubation (<6 h)	1.2%	1.9%	3.6%	.31	1.00
Prolonged ventilation (≥24 h)	4.9%	5.0%	14.6%	**.01**	.61
Discharged home	92.6%	90.1%	80.0%	**.02**	.21
RASS high	0.63	0.69	0.81	.28	.44
RASS low	−1.00	−1.02	−1.31	.07	.50
Depression	2.5%	10.6%	16.4%	**.05**	**.01**
Anxiety	4.9%	16.8%	20.0%	.17	**.005**
Smoker	33.3%	35.4%	29.1%	.43	.94
Alcohol use	13.6%	13.7%	9.1%	.36	.80
Glucose high	209.15	220.31	213.37	.76	.31
Glucose low	93.51	94.89	85.94	**.001**	.69
Sedation/pain/NMBD	9.9%	14.9%	14.6%	.80	.27
Valve only, valve + CAB, and other procedures: N = 100 patients					
N	36 (36%)	39 (39%)	25 (25%)		
Hospital LOS (d)	7.97	7.42	9.13	.28	.94
ICU LOS (d)	2.56	2.64	2.38	.77	.98
Mean ventilation time (h)	24.00	21.80	15.10	.36	.53
Early extubation (<6 h)	0.0%	0.0%	0.0%	1.00	1.00
Prolonged ventilation (≥24 h)	22.2%	15.4%	16.0%	1.00	.41
Discharged home	91.7%	89.7%	92.0%	1.00	1.00
RASS high	0.47	0.54	1.46	**<.001**	**.03**
RASS low	−0.86	−0.67	−0.79	.88	.89
Depression	5.6%	10.3%	4.0%	.68	1.00
Anxiety	13.9%	20.5%	8.0%	.34	.82
Smoker	16.7%	15.4%	4.0%	.18	.41
Alcohol use	20.0%	17.9%	24.0%	.78	.59
Glucose high	199.30	199.90	245.30	**<.001**	.15
Glucose low	90.40	95.60	86.90	.12	.61
Sedation/pain/NMBD	19.4%	12.8%	20.0%	.64	.63

Bold indicates stastically significant. *CAB*, Coronary artery bypass; *PICS*, post–intensive care syndrome; *LOS*, length of stay; *ICU*, intensive care unit; *RASS*, Richmond Agitation Sedation Scale; *NMBD*, neuromuscular blockade

Criteria for early extubation (<6 hours), prolonged ventilation (≥24 hours), and LOS measures were guided by Society of Thoracic Surgery definitions. The Richmond Agitation Sedation Scale (RASS) score used was the highest recorded during their ICU stay. Glucose values were included throughout the hospital encounter. All patients were treated according to our CS Enhanced Recovery standardized protocols that include a multimodal opioid-sparing medication regimen of ketamine infusion dexmedetomidine infusion and oral acetaminophen, and gabapentin with as-needed oxycodone. Standard glucose monitoring consisted of insulin infusion every hour in the operating room and ICU with transition to checks before meals and at bedtime with prandial and sliding scale coverage. Our glucose treatment aims for a glucose of 140 to 180 mg/dL.

## Results

A total of 397 patients were evaluated. Cases included coronary artery bypass surgery, valve surgery, and aortic surgery (Table 2). There were no significant differences in demographics (Table 3). Seventy percent of patients reported symptoms with mild (50%) or strong (20%) association with PICS. Two patients (0.5%) declined to fill out the survey. Nine patients (2%) did not fill out the form completely. Of the PICS domains, issues with behavioral symptoms were reported most frequently (64%) followed by functional symptoms (44%). The cognitive category had the lowest prevalence (23%). Patients who were more likely to show a strong positive PICS score when compared with the others surveyed were female (*P =* .04), White (*P =* .03), and requiring new dialysis (*P =* .01). A linear regression model with PICS score as an end point correlated with new dialysis during hospitalization (*P =* .002). A logistic regression model against high scoring PICS (score of 10+) also correlated with new dialysis (*P =* .018). A statistically significant correlation between hypoglycemia (*P <* .001) and high inpatient RASS scoring (*P =* .001) with strong positive PICS score was observed. Further, a history of diabetes (*P =* .05), depression (*P =* .01), and anxiety (*P =* .01) were more commonly observed in patients with PICS syndrome (Table 1). Patients undergoing isolated CABG were more likely to report symptoms compared with patients undergoing isolated valve, combination valve/CABG, or aortic surgery (73% vs 64%). The isolated CABG (n = 297) group that was found to have strong positive scores correlated with prolonged ICU LOS (*P =* .03), prolonged ventilation (*P =* .01), discharge to home rather than rehabilitation (*P =* .02), history of depression (*P =* .05), and hypoglycemia (*P* = .001). A history of anxiety and depression correlated with any level PICS symptoms. For the combination valve/CABG or aortic surgery patient group (n = 100), positive PICS scores were associated with hyperglycemia (*P <* .001) and high RASS (*P <* .001) score (Table 2).

**Table 3:** Patinet characteristics

PICS demographics: N = 397 patients	No PICS (0 survey points)	Weak positive (1-9)	Strong positive (10+)	*P* value (strong vs others)	*P* value (strong + weak combined vs no PICS)
Age	66.38	66.48	66.72	.82	.88
Female gender	24.8%	25.7%	38.4%	**.04**	.45
White race	80.5%	85.3%	72.6%	**.03**	.77
Black race	14.2%	12.0%	20.5%	.09	.95
Hispanic ethnicity	1.8%	1.1%	2.7%	.39	.86
Diabetes	36.3%	46.1%	50.7%	.20	**.05**
Chronic lung disease	16.8%	16.8%	24.7%	.12	.62
Hypertension	86.7%	90.6%	94.5%	.17	.14
Dialysis	0.9%	1.0%	5.5%	**.01**	.36
CVD	22.1%	25.1%	24.7%	.91	.55
Prior CVA	10.6%	7.3%	8.2%	.93	.33
EF	55.05	54.71	55.03	.87	.78
Nonelective status	61.9%	58.6%	57.5%	.72	.51
Mean STS PROM	1.6%	1.5%	1.7%	.48	.62

Bold indicates stastically significant. *PICS*, Post–intensive care syndrome; *CVD*, cardiovascular disease; *CVA*, cerebrovascular accident; *EF*, ejection fraction; *STS PROM*, Society of Thoracic Surgeons Predicted Risk of Mortality.

## Discussion

This prospective analysis demonstrated a high prevalence of PICS (70%) in the patients undergoing CS. Our analysis demonstrated a higher prevalence of PICS after CS in patients who are female and White, and who required new initiation of dialysis during hospitalization. Hypoglycemia and high RASS scores also correlated with high PICS scores. A trend was also found in patients with history of diabetes, depression, and anxiety. Unique to our study, White patients were more likely to report PICS symptoms, although the reasons behind this association remain unclear. We also found patients requiring new dialysis linked to high scoring PICS.

### Limitations

Limitations of this study include the fact that this is a single-center study with a relatively small cohort. Preexisting cognitive conditions were not excluded, and this could confound the findings. However, this was an inclusive, real-world, initial assessment of the prevalence of PICS after CS. Also, evaluations were only completed postoperatively and self-reported; thus, individual bias cannot be excluded. Some patients may have been unaware of their own cognitive deficits and therefore under-reported their impairment.^10^ There are limited published studies referencing the use of the HABC-M SR. We have initiated a follow-up study that includes the preoperative assessment of patients and follow-up for 1 year after CS.

## Conclusions

The gap in literature related to patients with PICS and CS suggests a need for more targeted studies to better understand the frequency, severity, and long-term effects. Our study demonstrated that there is a significant prevalence of PICS after CS. We identified female gender, White race, hemodialysis, hypoglycemia, and higher RASS scores as factors correlated with increased risk of PICS. A history of anxiety and depression correlated with any level PICS symptoms. We also found that patients undergoing isolated CABG were more likely to report PICS symptoms. This group correlated with prolonged ICU LOS, prolonged ventilation, discharge to home rather than rehabilitation, history of depression, and hypoglycemia. Further research is needed to confirm the findings of this analysis. Important areas of focus for future research include identifying patients at risk preoperatively, assessing duration of symptoms, and defining modifiable risk factors, especially those associated with CS.

## Conflict of Interest Statement

A.R.: Speaker's Bureau Edwards LifeSciences. R.C.A.: Honoraria from Edwards LifeSciences and HLS therapeutics. Ad Board for Renibus Therapeutics Inc. R.S.: Consulting/advisory relationships with Zimmer Biomet, AtriCure, La Jolla, Terumo, and Encare. All other authors reported no conflicts of interest.

The *Journal* policy requires editors and reviewers to disclose conflicts of interest and to decline handling or reviewing manuscripts for which they may have a conflict of interest. The editors and reviewers of this article have no conflicts of interest.
